# Analysis of lung stromal expression of the atypical chemokine receptor ACKR2 reveals unanticipated expression in murine blood endothelial cells

**DOI:** 10.1002/eji.201948374

**Published:** 2020-03-17

**Authors:** Christopher A.H. Hansell, Samantha Love, Marieke Pingen, Gillian J. Wilson, Megan MacLeod, Gerard J. Graham

**Affiliations:** ^1^ Chemokine Research Group Institute of Infection Immunity and Inflammation University of Glasgow Glasgow G12 8TA UK

**Keywords:** ACKR2, Alexa‐Fluor labeling, Blood endothelial cells, Lung, Pulmonary immunity

## Abstract

Analysis of chemokine receptor, and atypical chemokine receptor, expression is frequently hampered by the lack of availability of high‐quality antibodies and the species specificity of those that are available. We have previously described methodology utilizing Alexa‐Fluor‐labeled chemokine ligands as versatile reagents to detect receptor expression. Previously this has been limited to hematopoietic cells and methodology for assessing expression of receptors on stromal cells has been lacking. Among chemokine receptors, the ones most frequently expressed on stromal cells belong to the atypical chemokine receptor subfamily. These receptors do not signal in the classic sense in response to ligand but scavenge their ligands and degrade them and thus sculpt in vivo chemokine gradients. Here, we demonstrate the ability to use either intratracheal or intravenous, Alexa‐Fluor‐labeled chemokine administration to detect stromal cell populations expressing the atypical chemokine receptor ACKR2. Using this methodology, we demonstrate, for the first time, expression of ACKR2 on blood endothelial cells. This observation sets the lung aside from other tissues in which ACKR2 is exclusively expressed on lymphatic endothelial cells and suggest unique roles for ACKR2 in the pulmonary environment.

## Graphical abstract

Fluorescent‐CCL22 administration in vivo reveals pulmonary ACKR2 expression patterns. Intratracheal administration labels fibroblasts and some endothelial cells, while intravenous administration labels vascular endothelial cells. Our data provide the first evidence of ACKR2 expression on vascular endothelial cells. This is a versatile method applicable to analyzing other chemokine receptors in vivo.

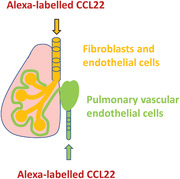

## Introduction

In vivo leukocyte migration is regulated, in the main, by proteins belonging to the chemokine family of chemotactic cytokines [1, 2]. This family is defined on the basis of a conserved cysteine motif in the mature sequence of its members and is divided into CC, CXC, XC, and CX3C subfamilies according to the specific configuration of this motif. The chemokine family arose early in vertebrate evolution [3] (prevertebrate species do not have chemokines) and the primordial chemokine was almost certainly CXCL12, which plays essential roles in stem cell migration during embryogenesis [4, 5, 6, 7, 8, 9]. From this one chemokine and its receptor CXCR4, through gene duplication, the family has expanded to the point at which mammals have approximately 45 chemokines, and 18 signaling chemokine receptors, which together orchestrate in vivo homeostatic and inflammatory leukocyte migration. Chemokine regulation of cellular migration is extremely complex and, particularly in the case of inflammation [10], poorly understood, which has contributed to ongoing problems in therapeutically targeting inflammatory chemokine receptors in immune and inflammatory diseases [11].

In addition to signaling chemokine receptors that belong to the G‐protein‐coupled receptor family [12], chemokines also bind to a subfamily of atypical chemokine receptors (ACKRs), which are generally stromally expressed and which fine‐tune in vivo chemokine activity by scavenging chemokines and therefore regulating chemokine availability [13, 14, 15]. There are currently four members of the ACKR family: ACKR1 (formerly known as DARC), ACKR2 (formerly known as D6), ACKR3 (formerly known as CXCR7), and ACKR4 (formerly known as CCX‐CKR). With the exception of ACKR1, these receptors exhibit spontaneous internalization and recycling activity and scavenge chemokines from the environment and target them for lysosomal degradation. ACKR3 carries out this role in some essential developmental contexts and is strongly evolutionarily conserved [5, 6, 16, 17]. ACKR4 scavenges chemokines within the LN to generate intra‐LN gradients and facilitate DC migration from the subcapsular sinus into the T‐cell zone of the LN [18]. We have had a particular interest in ACKR2, which is the prototypic member of the ACKR family [19]. This receptor binds, internalizes, and degrades all inflammatory chemokines belonging to the CC‐chemokine subfamily and thus plays an essential role in the resolution of the inflammatory response [20, 21, 22, 23, 24]. This has implications for tumorigenesis [25, 26, 27, 28] as well as for branching morphogenesis in a number of developmental contexts [29, 30]. ACKR2 is predominantly expressed on lymphatic endothelial cells [31, 32] and placental trophoblasts [23, 33, 34] with expression also being detected on subsets of splenic B cells [35]. The ACKRs are therefore central regulators of chemokine activity in vivo.

One of the challenges in studying chemokine receptors, including ACKRs, is access to high‐quality antibodies. While antibodies to some typical and atypical receptors are available, it has proven extremely difficult to raise useful antibodies to others. For example, there are no high‐quality antibodies available for detection of murine ACKR2. A further issue is that even for receptors for which antibodies are available, these antibodies are invariably species specific and it is therefore difficult to carry out analyses in either nonmurine or nonhuman species. This is particularly important for highly conserved receptors such as ACKR3. The availability of a generic methodology to allow detection of ACKRs (and amenable to detection of typical receptors), which would be usable in vivo and applicable to numerous species, would therefore represent a significant technological development.

Here, we describe a novel methodology for the detailed analysis of the phenotypes of ACKR2‐expressing cells in lung stroma. The technology is conceptually similar to that reported by Ameti and colleagues [36] and uses ACKR2‐dependent internalization of a fluorescently tagged ligand to identify receptor expressing cells and we demonstrate the utility of comparing intratracheal and intravenous administration in defining, and discriminating between, discrete receptor expressing stromal cell types. Using this approach, we report, for the first time, robust expression of ACKR2 by pulmonary blood endothelial cells. This has not been reported in any other tissues and suggests unique roles for ACKR2 in lung function and pulmonary immunity.

## Results

### ACKR2 is stromally expressed in the lung

**Figure 1:**
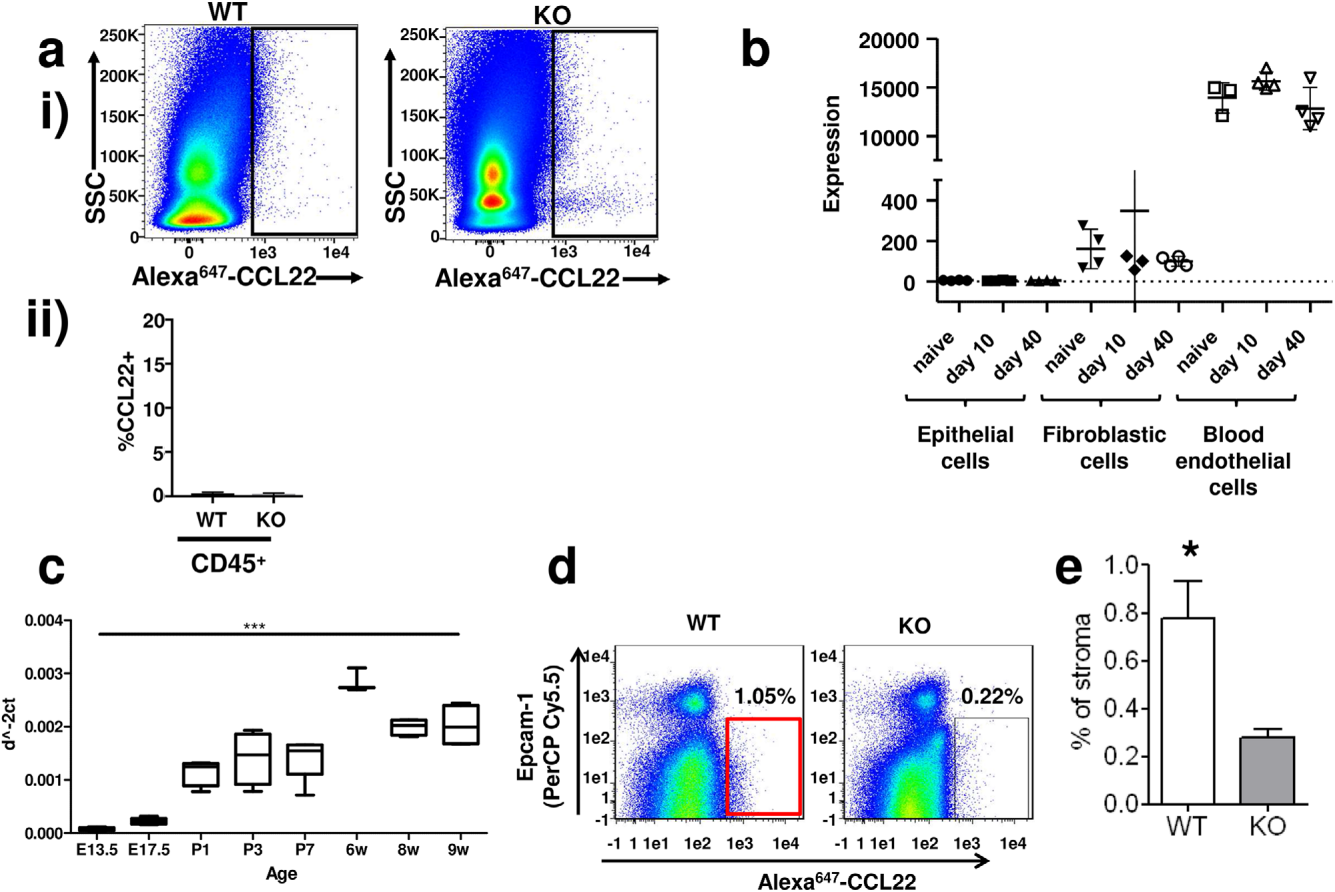
In vitro analysis of pulmonary ACKR2 expression. (a) (i) Flow cytometric analysis showing lack of expression of ACKR2 by CD45+ve cells in lung digests as assessed by comparing Alexa‐CCL22 uptake (*X*‐axis) in WT and ACKR2^−/−^ (KO) lungs. The *Y*‐axis shows side scatter (SSC) (*n* = 3 WT and 3 KO mice per experiment. The experiment was performed three times). (ii) Graph showing combined results of (i) for multiple analyses of Alexa‐CCL22 binding by CD45^+^ cells from the lung. There are no significant differences in the binding profiles of WT and ACKR2^−/−^ (KO) CD45^+^ cells. Error bars represent mean± SD. (b) Analysis of transcriptomic data generated from lung stromal populations showing low level ACKR2 expression in fibroblasts but high‐level expression in blood endothelial cells (analyzed using a bespoke bioinformatics pipeline available through Glasgow Polyomics:www.polyomics.gla.ac.uk). These data were generated from resting lung stromal populations as well as from the same populations retrieved from lungs at the indicated times (10 and 40 days) postinfluenza virus infection. (Data were generated and combined using RNA from four separate lung cell harvests and the transctiptomic analyses performed once on these samples. Error bars represent mean ± SD. (c) qPCR analysis of pulmonary ACKR2 expression from embryonic day E13.5 to adult 9‐week‐old mice. Data were normalized to expression of the housekeeping gene Tata‐binding protein. Statistical significance was tested using a one‐way ANOVA test and ***p < 0.001. (Each time point represents PCR data combined from four separate lung preps. PCR was performed on one occasion once all samples were obtained.) Error bars represent mean ± SD. (d) Flow cytometric analysis of CD45‐ve Alexa‐CCL22 internalizing cells from WT and ACKR2^−/−^ lungs. The *X*‐axis shows the Alexa‐CXCL22 binding, while the *Y*‐axis shows staining for the epithelial marker EpCAM. (*n* = 3 WT and 3 KO mice per experiment. The experiment was performed three times.) (e) Quantification of the percentage of ACKR2+ve stromal cells detected using this Alexa‐CCL22 in vitro labeling approach. Data are combined from three experiments with *n* = 3 mice/group. Statistical significance was tested using Student's *T* test and *p < 0.05. Error bars represent mean ± SD.

Data on ACKR2 expression profiles available through the Immgen database (www.immgen.org) reveal that the lung is the tissue with the highest expression (Supporting Information Fig. 1). We have previously described the versatile use of fluorescently labeled chemokines, instead of antibodies, to detect their cognate receptors using flow cytometry as a read out [35, 37‐39]. We used this approach with Alexa‐647‐labeled CCL22 (a high‐affinity ACKR2 ligand: Alexa‐CCL22) uptake to detect ACKR2 in lung digests. Importantly, CCL22 also binds to the chemokine receptor CCR4 so to control for any contribution of this receptor to cellular CCL22 binding, and to confirm specific binding to ACKR2, we have included ACKR2^−/−^ mice and tissues in all our analyses. Comparing CCL22 binding in WT and ACKR2^−/−^ tissues and cells therefore allows us to specifically determine the expression patterns of ACKR2. Importantly, there are no differences in circulating levels of CCL22 between WT and ACKR2^−/−^ mice, which might confound these analyses (Supporting Information Fig. 2). Further reasons for selecting CCL22 for this analysis include the fact that this ligand does not display the broad receptor binding profiles of other ACKR2 ligands and expression of CCR4 is more limited than the receptors that bind the other ACKR2 ligands [14]. Flow cytometric analysis of CD45^+^ cells from Alexa‐CCL22‐stained lung digests failed to detect any significant expression on CD45^+^ leukocytes in either WT or ACKR2^−/−^ lungs (Fig. 1a). The exemplar flow cytometry plot shown in Fig. 1a(i) revealed a low level of Alexa‐CCL22 binding to CD45^+^ cells in ACKR2^−/−^ lung digests, which is not seen in WT digests. We did not see this routinely and repeated flow cytometric analyses (Fig. 1a(ii)) revealed essentially undetectable Alexa‐CCL2 binding by either WT or ACKR2^−/−^ CD45^+^ cells. These data, therefore, indicated that ACKR2 expression in the lung was predominantly stromal in origin. We used RNA sequencing to generate data on the transcriptional profile of FACS‐sorted pulmonary stromal cell types at rest and over the course of an influenza infection to examine possible pathogen‐driven alterations in expression. As shown in Fig. 1(b), ACKR2 expression was essentially undetectable in epithelial cells but was present at low levels in fibroblasts and at very high levels in blood endothelial cells. Expression did not vary significantly over the course of influenza infection. Examination of pulmonary ACKR2 expression by qPCR from embryonic day 13.5 to 9 weeks of age indicated that expression is low within the embryo but that it is markedly upregulated immediately after birth and presumably coincident with the onset of breathing. This increased level is maintained and increased as mice age (Fig. 1c).

Next, we tried to use the in vitro Alexa‐CCL22 detection method to examine ACKR2 expression on nonleukocytic stromal cells in the digested lung. However, and as shown in Fig. 1d, this technology, which works well with leukocytes [35, 39, 40] and mammary gland fibroblasts [30], revealed only a minor difference in the numbers of Alexa‐CCL22 internalizing cells in WT or ACKR2^−/−^ lungs. These data are summarized numerically in Fig. 1e.

Overall, therefore, these data indicate that ACKR2 is predominantly expressed on stromal cells within the lung but that flow cytometry utilizing fluorescent chemokine uptake with digested lung tissue has limited sensitivity to detect the key stromal expressing cell types. Importantly, the Alexa‐CCL22 was added to the lung cells after digestion was completed and the cells washed. There is therefore unlikely to be any contribution of proteolytic degradation of the ligand to the weak binding results obtained in these experiments.

### Intratracheal fluorescent chemokine administration detects key ACKR2‐expressing stromal components

**Figure 2:**
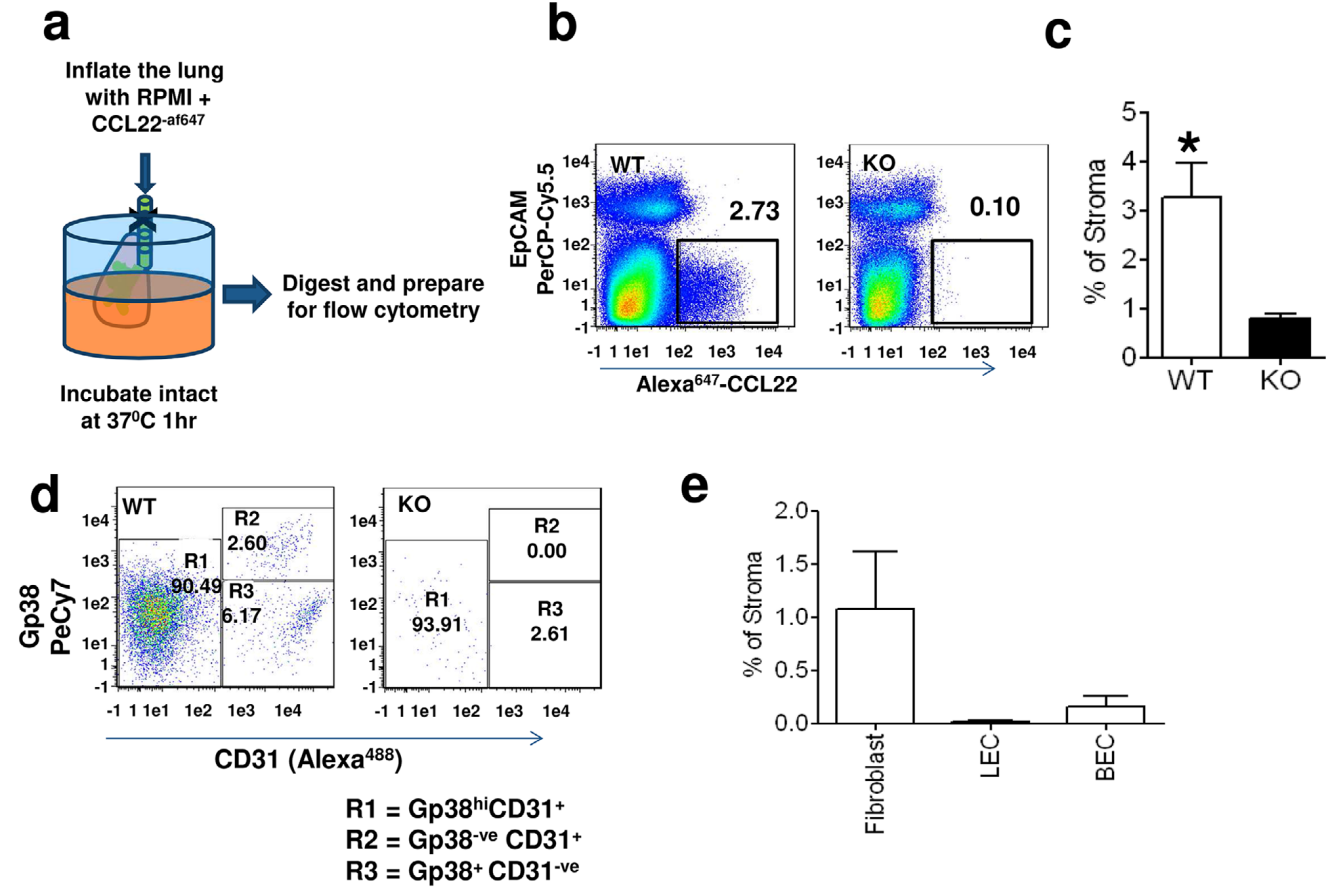
Detection of stromal ACKR2 expression by intratracheal administration of Alexa‐CCL22. (a) Diagram indicating the basic methodology that involves removing intact lungs and inflating them through the trachea using an RPMI/Alexa‐CCL22 mix. The lung is then incubated at 37°C for 1 h, then digested and prepared for flow cytometry. (b) Flow cytometric analysis showing the presence of a significant Alexa‐CCL22 internalizing stromal component in the lung. The *X*‐axis shows the Alexa‐CXCL22 binding/internalization, while the *Y*‐axis shows staining for the epithelial marker EpCAM. (*n* = 3 WT and 3 KO mice per experiment. The experiment was performed three times.) (c) Quantification of the levels of stromal ACKR2 detected in WT and ACKR2^−/−^ (KO) lungs following intratracheal Alexa‐CCL22 administration. Data are combined from three experiments with *n* = 3 mice/group. Statistical significance was tested using Student's *T* test and *p < 0.05. Error bars represent mean + SD. (d) Flow cytometric analysis of the CD45‐ve EpCAM‐ve nonepithelial stromal component expressing ACKR2 on the basis of CD31 and Gp38 expression. Data shown are from Alexa‐CCL22 binding lung stromal cells from WT (left plot) and ACKR2^−/−^ (KO; right plot) mice. (*n* = 3 WT and 3 KO mice per experiment. The experiment was performed three times.) (e) Summary analysis of the percentage of CD45‐ve stroma associated with each of the indicated cell types following intratracheal administration of Alexa‐CCL22. Data are combined from three experiments with *n* = 3 mice/group. Error bars represent mean ± SD.

We reasoned that the function of stromal ACKR2 expression may be dependent on interactions with other stromal components and that the inability to detect it using flow cytometry reflects the inability to take up Alexa‐CCL22 due to absence of these interactions. We therefore harvested intact lungs and inflated them with Alexa‐CCL22 (in RPMI) intratracheally followed by incubation at 37°C for 1 h (Fig. 2a). Following this, flow cytometric analysis of digested lungs focusing on CD45‐ve cellular populations now revealed a sizeable population of Alexa‐CCL22 internalizing cells in WT lungs, which are absent from ACKR2^−/−^ lungs (Fig. 2b). These cells are nonepithelial as they are negative for EpCAM staining and these data are summarized numerically in Fig. 2c. Further flow cytometric analysis indicated that these cells fall into three basic categories as defined by CD31 and Gp38 expression (Fig. 2d). These represent fibroblasts (R1; CD31−Gp38−), lymphatic endothelial cells (R2; CD31+Gp38+), and blood endothelial cells (R3; CD31+Gp38−), which are enumerated as shown in Fig. 2e.

Overall, these data demonstrate that it is possible to detect stromal cell populations that bind and internalize Alexa‐CCL22 via ACKR2 by introducing the chemokine intratracheally into the intact lung. They also demonstrate novel stromal expression patterns for ACKR2 within the lung.

### A subpopulation of fibroblasts in the lung expresses ACKR2

**Figure 3:**
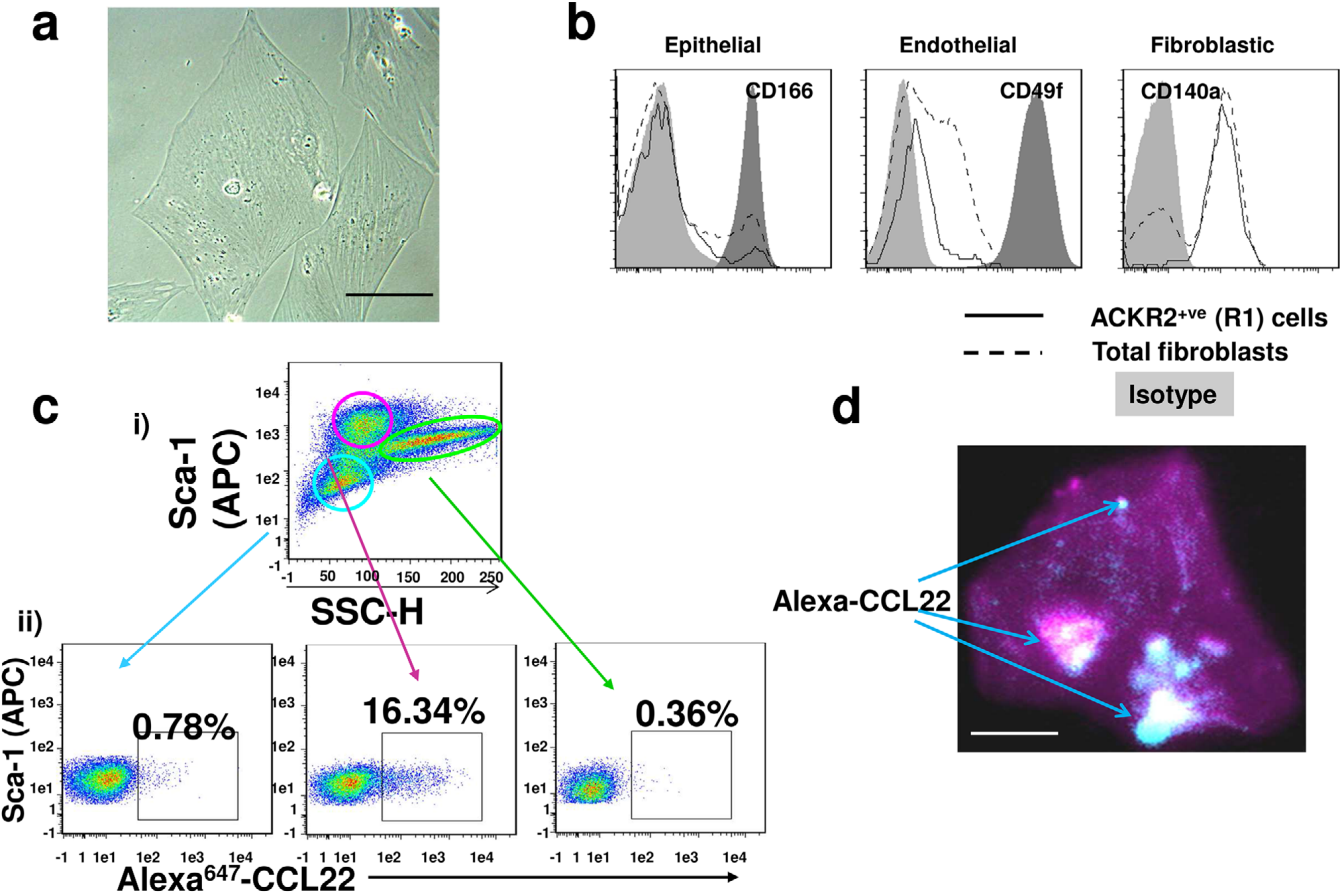
Identification of a fibroblastic population as a component of the ACKR2‐expressing stromal population. (a) Brightfield image of cells sorted for internalization of fluorescently labeled Alexa‐CCL22. These cells are negative for Gp38, CD45, and CD31 expression (*n* = 3). (Representative image from three separate in vitro cultures.) Image captured at 63× magnification. Scale bar: 10 µm. (b) Flow cytometric analysis of the fibroblastic population showing absence of expression of epithelial (CD166) and endothelial (CD49f) markers but positive expression of fibroblastic markers (CD140a). (*n* = 3 mice per experiment. The experiment was performed three times.) (c) (i) Flow cytometry profile showing Sca‐1 staining and SSC for the CD140a^+^ fibroblast population. (ii) Flow cytometric analysis of Alexa‐CCL22 binding by each of the 3 Sca‐1+ve populations. (*n* = 3 WT and 3 KO mice per experiment. The experiment was performed three times.) (d) Fluorescent confocal image of ACKR2+ve fibroblastic cells binding and internalizing Alexa‐CCL22 (arrowed). (Representative image from three separate in vitro cultures). Image captured at 63× magnification. Scale bar: 10 µm.

Cells identified in the R1 gate in Fig. 2d, which were negative for markers of lymphatic and vascular endothelial cells, were further phenotyped. Initially, these cells were isolated by cell sorting and then grown in tissue culture. As shown in Fig. 3a, the cells display a morphology suggestive of a fibroblastic phenotype. Flow cytometric analysis revealed that these cells are negative for markers of epithelial (CD166) and endothelial (CD49f) cells but positive for the fibroblastic marker CD140a (Fig. 3b). The CD140a+ve cells can be subdivided into three populations based on Sca‐1 staining and SSC. Further Alexa‐CCL22 binding assays revealed that the dominant ACKR2‐expressing population was Sca‐1 high and SSC lo/mid in phenotype (Fig. 3c). Figure 3d further shows that sorted ACKR2+ fibroblastic cells were capable of binding Alexa‐CCL22 as indicated by the extensive fluorescence (arrows), confirming expression of functional ACKR2 (Fig. 3b). Given the punctate nature of much of this staining, we propose that it is largely intracellular in nature. Overall these data indicate that one of the ACKR2‐expressing stromal components in the lung detected by intratracheal Alexa‐CCL22 administration is a fibroblastic subpopulation characterized by CD140a and Sca‐1 expression.

### Intravenous fluorescent chemokine administration detects further ACKR2‐expressing stromal components

**Figure 4:**
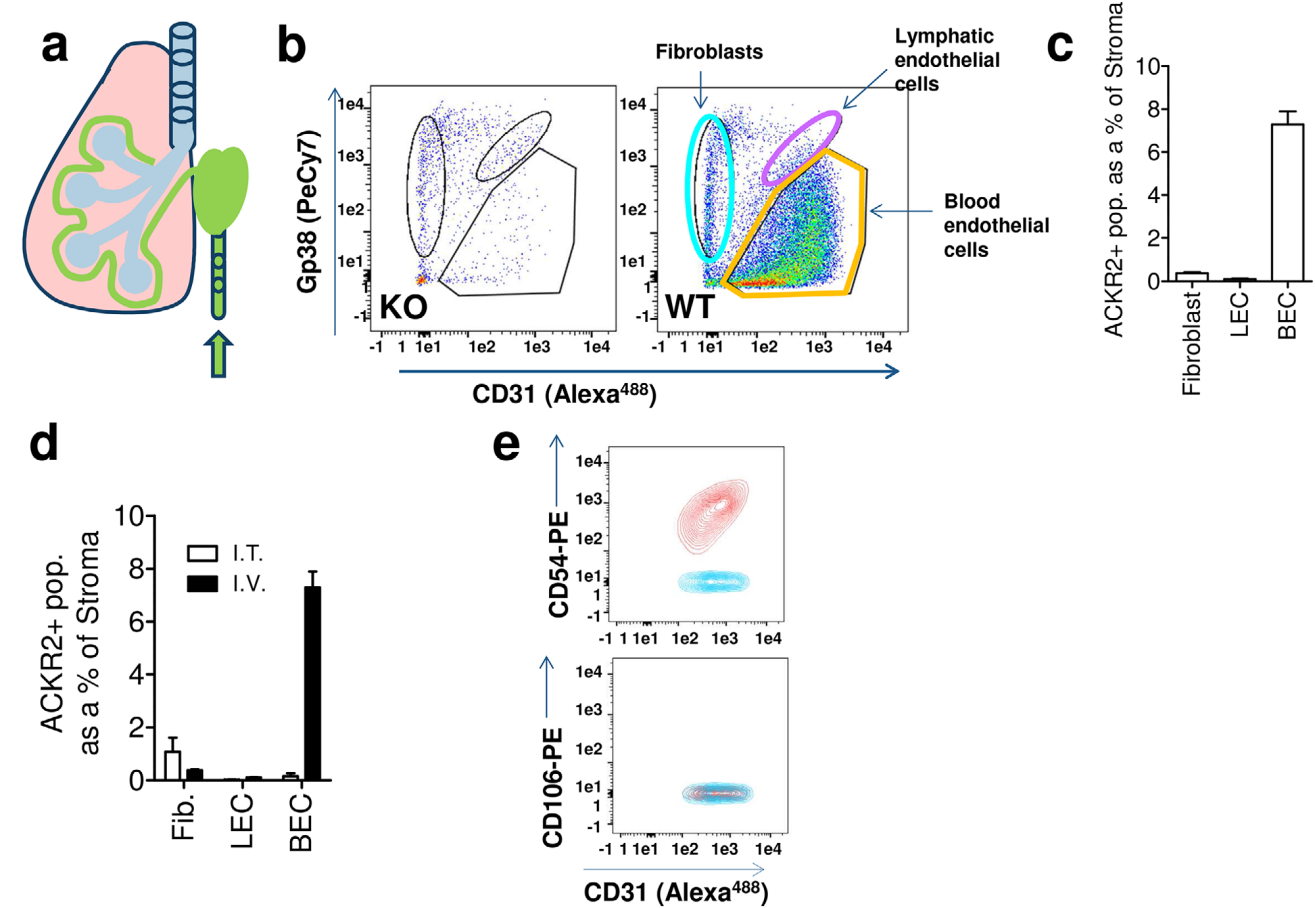
Intravenous administration of Alexa‐CCL22 reveals strong expression of ACKR2 on the luminal face of pulmonary vascular endothelial cells. (a) Diagram indicating intravenous injection of Alexa‐CCL22 into live mice. (b) Flow cytometric analysis of CD31 (*X*‐axis) and Gp38 (*Y*‐axis) expression by CD45‐ve EpCAM‐ve stromal cells that internalize Alexa‐CCL22 in ACKR2^−/−^ (KO) and WT lungs. The identified populations are indicated. (*n* = 3 WT and 3 KO mice per experiment. The experiment was performed three times.) (c) Quantification of the % of CD45‐ve stromal cellular populations internalizing Alexa‐CCL22 following intravenous administration of Alexa‐CCL22. Data are combined from three experiments with *n* = 3 mice/group. Error bars represent mean ± SD. (d) Comparison of intratracheal and intravenous administration and the impact on the relative size of the ACKR2+ stromal cell populations detected by flow cytometry. Fib., fibroblasts; LEC, lymphatic endothelial cells; BEC, blood endothelial cells. Data are combined from three experiments with *n* = 5 mice/group. Error bars represent mean ± SD. (e) Flow cytometric analysis of the CD31+ve, Alexa‐CCL22 internalizing blood endothelial cells indicate that they are positive for expression of CD54 and negative for CD106 and thus are alveolar, rather than peribronchial, endothelial cells. Red: the gated CD31^+^ cells that have been antibody stained; blue, fluorescence minus one (FMO) control. (*n* = 5 mice per experiment. The experiment was performed twice.)

We have previously reported expression of ACKR2 in lymphatic endothelial cells [31], however expression in blood endothelial cells as shown in Fig. 2d and e has not been reported. In fact, and as shown in Supporting Information Fig. 3, comparison of transcriptomic data from a broad range of microvascular endothelial types indicates that expression in blood endothelial cells is peculiar to the lung. Given that we have reported polarization of expression of ACKR2 in trophoblasts and lymphatic endothelial cells [31, 34, 41], we wondered whether it may also be polarized in blood endothelial cells. If this is the case, then it is possible that the vascular facing aspect of endothelial cells may be more able to internalize Alexa‐CCL22 and thus the blood endothelial cell component might be underestimated by the intratracheal administration of the chemokine. We therefore next injected Alexa‐CCL22 intravenously (Fig. 4a) and then harvested the lung for flow cytometric analysis of Alexa‐CCL22 cellular interactions. Again, this analysis revealed that Alexa‐CCL22 was not bound by lung‐resident CD45^+^ cells (data not shown) but uptake was seen in three populations of nonepithelial stromal cells in WT but not ACKR2^−/−^ lungs. These populations comprised fibroblasts, blood endothelial cells, and lymphatic endothelial cells (Fig. 4b). However, and in contrast to intratracheal administration, intravenous administration detected blood endothelial cells as being by far the dominant ACKR2‐expressing stromal population. Figure 4c shows that intravenous administration highlights blood endothelial cells as being the predominant stromal cell component in the lung and comparing intratracheal administration with intravenous administration (Fig. 4d) reveals the differences in cellular detection using these two approaches. As we have previously shown expression of ACKR2 by lymphatic endothelial cells [31, 32], it remained possible that what we have characterized as lung blood endothelial cells are in fact lung lymphatic endothelial cells displaying an altered CD31/Gp38 phenotype compared to other lymphatic endothelial cell populations. To formally test this, we examined lung lymphatic endothelial cell expression of CD31 and Gp38 using Prox‐1 reporter mice. Prox‐1 is a definitive marker and an essential master regulator of lymphatic endothelial cells [42]. As shown in Supporting Information Fig. 4, Prox‐1+ve cells from reporter mouse lungs were exclusively co‐positive for CD31 and Gp38, confirming the faithfulness of the lymphatic phenotype in the lung. These data further confirm the blood endothelial nature of the ACKR2+ve stromal cells.

In the lung, there are two major blood vascular beds: one associated with bronchial tissues and one with alveolar tissues. These can be discriminated on the basis of CD54 and CD31 expression [43]. As shown in Fig. 4e, the Alexa‐CCL22 internalizing blood endothelial cell population is strongly co‐positive for CD31 and CD54, demonstrating that this population is associated with alveolar blood vessels and not peribronchial blood vessels.

### In situ hybridization and antibody expression confirm blood endothelial cell expression of ACKR2

**Figure 5:**
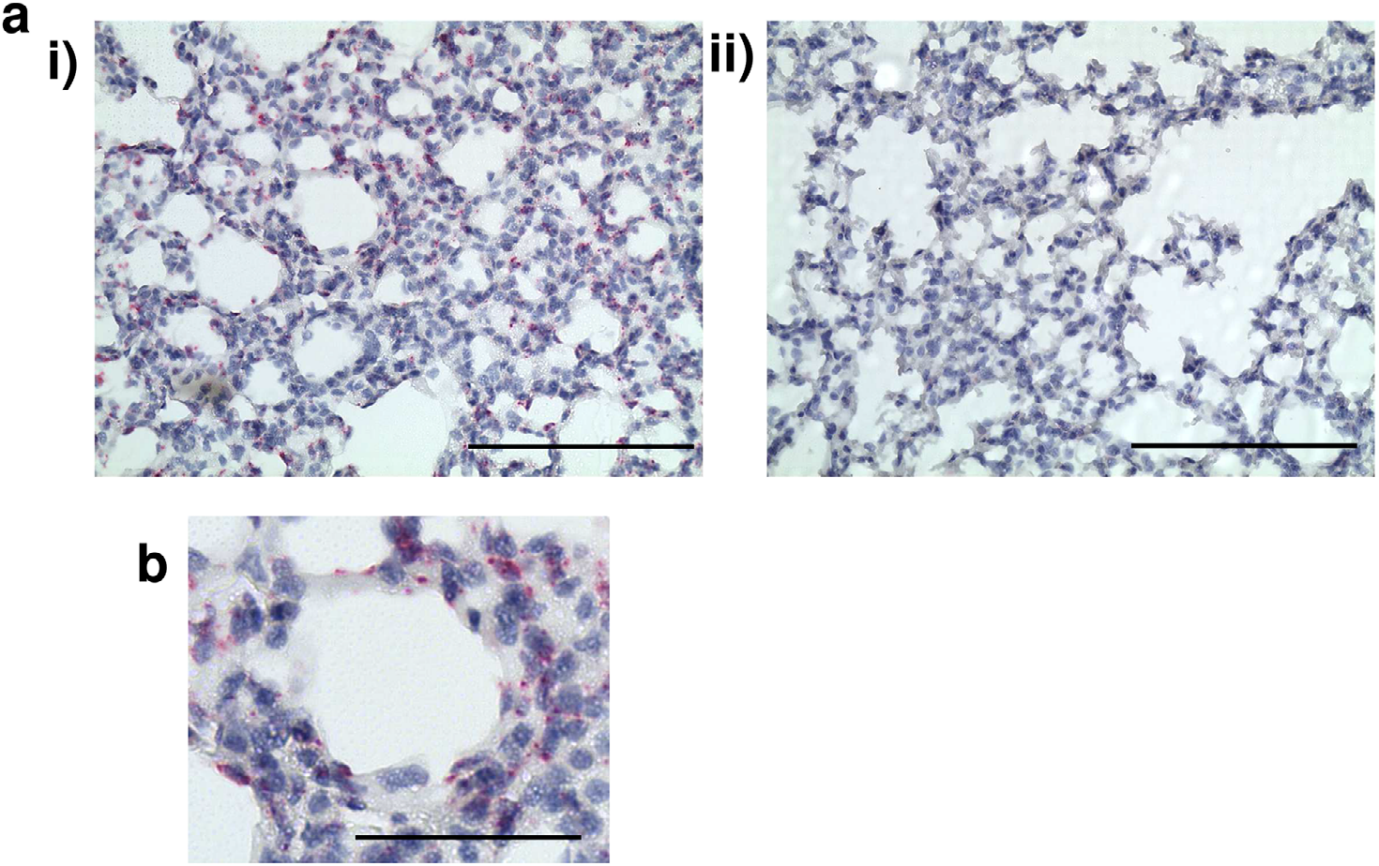
In situ and antibody detection of ACKR2 on pulmonary alveolar blood endothelial cells in mouse and human lung. (a) Representative in situ hybridization showing positivity for ACKR2 expression in alveolar endothelial cells in murine lungs (arrow). (i) WT lungs and (ii) ACKR2^−/−^ lungs. Note the absence of signal in alveolar macrophages. Image captured at 10× magnification. Scale bars: 200 µm. (*n* = 3 mice per experiment. The experiment was performed twice). (b) High power image cropped from (i) showing the peri‐alveolar localization of the vascular staining. This image has been amplified 2× and the scale bar = 100 µm.

To further validate ACKR2 expression by murine pulmonary vascular endothelial cells, we carried out in situ hybridization. As shown in Fig. 5a, clear in situ hybridization signals were seen associated with alveolar endothelial cells in blood vessels surrounding the alveolar air space in WT, but not ACKR2^−/−^, adult murine lungs. A higher magnification image of a portion of Fig. 5a(i) is shown in Fig. 5b, further highlighting the peri‐alveolar localization of the vascular staining. Therefore, in situ hybridization confirms blood endothelial cell expression of ACKR2 in the murine lung.

## Discussion

ACKRs are predominantly expressed on stromal cell types and serve key functions in localizing and fine‐tuning chemokine activities [15]. While a number of antibodies and reporter mouse‐based approaches are available for analysis of ACKR expression patterns, in many cases these are limited and applicable only to mouse and humans. Given that many of the ACKRs display strong evolutionary conservation, other more versatile approaches would therefore represent a significant improvement in the methodological repertoire for ACKR expression analysis. Here, we demonstrate, using the lung, that fluorescently labeled chemokines can be used, with intact tissues, to precisely isolate and phenotypically define stromal cell types expressing individual ACKRs. This is particularly important when, as in the current analyses, removal of the cells from their stromal environment impairs ACKR2 function and thus frustrates this detection methodology. The ability to chemically synthesize chemokines with relative ease and to introduce discrete fluorescent markers at the carboxy terminus [40] means that this approach has full versatility and is appropriate for all members of the chemokine receptor family and all species expressing either typical or ACKRs. Importantly, while we demonstrate the utility of this approach using WT and KO mice, the use of appropriate unlabeled competing chemokines specific for the receptor being studied will allow this technology to be used under circumstances, or in species, where KO models do not exist.

Here, we show that intratracheal, and intravenous, administration of fluorescently labeled CCL22 is capable of identifying stromal cell populations expressing ACKR2. Importantly, in the context of vascular endothelial cell expression, intravenous administration has the advantage of detecting chemokine receptor expression with a polarity favoring expression on the luminal side of the endothelium. Together these approaches allowed us to define fibroblasts, lymphatic endothelial cells, and surprisingly alveolar blood endothelial cells as key sites of stromal ACKR2 expression in the lung. Notably, and in contrast to previous reports, we did not detect ACKR2 activity on alveolar macrophages [44, 45]. This may be due to species differences in expression or alternatively may be a consequence of nonspecific antibody internalization by the alveolar macrophages. Further analysis is required to address this discrepancy.

In summary, therefore we report a methodology appropriate for detecting ACKR expression in the lung, which we believe to be sufficiently versatile to be useful to detect other atypical and typical chemokine receptors in numerous tissues in vivo in divergent species. The data indicate for the first time the stromal expression patterns of ACKR2 in the lung with notably high expression levels on alveolar endothelial cells. These data will help interpret the outcome of analysis of ACKR2 function in the lung, which remains poorly defined.

## Materials and Methods

### Mice

Animal experiments were performed using cohoused mice in ventilated cages in a barrier facility that conformed to the animal care and welfare protocols approved by the University of Glasgow under the revised Animal (Scientific Procedures) Act 1986 and the European Union Directive 2010/63/EU. Ackr2‐deficient mice^21^ were bred in‐house (C57BL/6 background); wild‐type (WT) C57BL6/J mice were from Charles River Research Models and Services. Prox‐1 reporter mice were obtained from Jackson laboratories. All experimental mice were sex and age matched.

### qPCR

RNA was extracted using RNeasy columns with DNase treatment (Qiagen), and the amount of RNA was quantified on a Nanodrop 1000 Spectrophotometer (Thermo Fisher Scientific). cDNA was synthesized using High‐capacity RNA‐to‐cDNA kit by Applied Biosystems (Thermofisher). For all qPCRs, a final concentration of 0.2 mM primers was used for each PCR set up using PerfeCTa SYBR Green FastMix and ROX qPCR Master Mix (Quanta BioSciences). qPCRs were performed on a Prism 7900HT Fast Real‐Time PCR System (Applied Biosystems). The thermal cycles for qPCR of TBP and ACKR2 were 95˚C (3 min) for one cycle and 95˚C (3 s) and 60˚C (30 s) for 40 cycles. Relative expression was calculated using serial dilutions of cDNA standards. Primer sequences designed for qPCR and for producing cDNA standards were designed using Primer3 software (http://frodo.wi.mit.edu/cgi-bin/primer3/primer3_www.cgi). The following primers were used: mouse ACKR2, 5′‐TTCTCCCACTGCTGCTTCAC‐3′, 5′‐TGCCATCTCAACATCACAGA‐3′; mouse TBP primer: 5′‐ AAGGGAGAATCATGGACCAG‐3′, 5′‐CCGTAAGGCATCATTGGACT‐3′.

### In situ hybridization

Mice were culled using increasing concentration of CO_2_. Lungs were placed in 10% neutral buffered formalin at room temperature for 24–36 h before they were processed by dehydration using rising concentrations of ethanol, xylene stabilization, and paraffin embedding (Shandon citadel 1000; Thermo Shandon). Tissue was then sectioned onto Superfrost plus slides (VWR) at 6 µm using a Microtome (Shandon Finesse 325 Microtome; Thermo). All slides for analysis were processed together. Slides were baked at 60°C for 1 h before pretreatment. Slides were deparaffinized with xylene (5 min × 2) and dehydrated with ethanol (1 min × 2). In situ hybridization was performed using the RNAscope® 2.5 HD Reagent Kit‐RED from Advanced Cell Diagnostics (cat. no. 322350) and according to the manufacturer's instructions. Briefly, tissues were incubated with hydrogen peroxide for 10 min at RT. The slides were boiled in antigen retrieval buffer for 15 min. Slides were treated with “protease plus” for 30 min at 40°C. Slides were then hybridized using the RNAScope 2.5 Red Manual Assay (Advanced cell diagnostics) according to manufacturer's instructions using the Mm‐ACKR2 probe (NM_021609.4). Slides were mounted in DPX (Sigma Aldrich) and imaged on an EVOS M7000 microscope (Thermofisher).

### Intratracheal and intravenous chemokine administration

To administer fluorescent chemokine intratracheally, mice were euthanized using an appropriate schedule 1 method or CO_2_ exposure. The mice were then carefully dissected to remove the ribcage and expose the intact lungs and trachea in situ. Using a pair of surgical scissors, a small incision was made at the top of the exposed trachea toward the base of the jaw. A 2 µg/mL solution of Alexa 647^TM^ labeled CCL22 (Almac; Alexa‐CCL22) dissolved in RPMI/25mM HEPES was prepared in a polypropolene tube and preserved from light at room temperature until needed. Once the dissection was complete, a syringe with a 19G needle and loaded with 400 µL of the Alexa‐CCL22 solution was then inserted into the exposed trachea via the incision. The needle should be tight within the trachea and care should be taken not to pierce the trachea further down. The lungs were then inflated with the chemokine solution and the trachea carefully tied off with surgical thread to prevent the leakage of chemokine solution as the syringe is removed. The intact inflated lungs were then removed and placed into a falcon tube containing enough RPMI to cover the intact inflated lungs. The lungs were then incubated in a water bath for 1 h at 37°C. Following this time, the lungs were removed from the water bath and the surgical thread cut to allow draining of the remaining chemokine solution. The lungs were then digested for a single cell suspension as per the protocol.

### Flow cytometry

All flow cytometry analyses adhered to published guidelines for use of flow cytometry and cell sorting in immunological studies [46]. Lungs were removed and finely chopped with scissors, then incubated in digestion mix (1.6 mg/mL Dispase [Roche], 0.2 mg/mL Collagenase P [Roche], and 0.1 mg/mL DNase I [Invitrogen]) in HBSS on a gentle shake at 37°C for 40 min. Lungs were passed through a 40 µm mesh (Greiner Bio‐One), RBCs were lysed in 1 mL RBC lysis buffer (eBioscience) for 1 min, and then in 10 mL FACs buffer (PBS, 1% FCS, 0.02% sodium azide, 5 mM EDTA) to quench the reaction. The resulting single cell suspension was preincubated with Fc block (BD Biosciences) in FACS buffer (PBS, 1% FCS, and 5 mM EDTA) and labeled with fluorescent anti‐mouse Abs, including CD45 (30‐F11), Gp38 (8.1.1), CD140a (APA5), EpCAM‐1 (G8.8), CD31 (390), and CD49f (GoH3), each labeled with various fluorochromes (Biolegend) CD166 (eBioALC48) (Ebioscience). Dead cells were labeled using Fixable viability dye efluor 506 or efluor 780 (ebioscience). Cells were stained for 20 min on ice before washing with FACS buffer. Cells were either analyzed by flow cytometry immediately or fixed for 20 min with 2% methanol‐free paraformaldehyde (Invitrogen) before washing and placing at 4°C in the dark until ready to be analyzed typically 12–24 h later. Cells were analyzed using LSR2 flow cytometers (Beckton Dickinson) or sorted on a FACSAria. Data were analyzed using FlowJo Version 9.2 software (TreeStar) with populations defined by size, viability, and “fluorescence minus one” isotype controls. Gating strategies are shown in Supporting Information Fig. 5.

### Immunofluorescence

Alexa‐CCL22 labeled ACKR2+ fibroblasts were flow‐sorted from a single cell suspension using the ARIAII (Beckman and Dickinson) as per the flow protocol. ACKR2^+^ fibroblasts were resuspended in PBS at a density of 2000 cells/mL. Note that 100 µL of this cell suspension was loaded into a cytospin 3 machine (Thermo‐Shandon) and the cells were spun for 5 min at 200 rpm onto Superfrost^TM^ plus slides. The cells were air dried in the dark and mounted in Vectashield Hard set mounting medium (Vector Laboratories). The cells were visualized using a Zeiss Spinning Disc confocal microscope.

### Fibroblast culture and analyses

Primary ACKR2 positive fibroblasts were flow‐sorted from a single cell suspension using the ARIAII (Beckman and Dickinson) as per the flow protocol. Retrieved cells were spun down at 400*g* for 5 min and washed three times into EMEM with 15% FBS, 1× penicillin/streptomycin, nonessential amino acids, and sodium pyruvate. The cells were plated at a density of approximately 5 × 10^3^ cells per well of a 24‐well plate (Gibco). Fibroblasts were cultured as standard in an incubator at 37°C, 5% CO_2_.

### Statistics

Statistical tests were carried out using Graph Pad Prism software and the individual tests used are indicated in the relevant figure legends. *P* = 0.05 was taken as a cut‐off for statistical significance.

## Conflict of interest

The authors declare no financial or commercial conflict of interest.

**Abbreviation**
ACKR: atypical chemokine receptor

## Supporting information

Supporting Information

## References

[1] Rot, A. and von Andrian, U. H. , Chemokines in innate and adaptive host defense: basic chemokinese grammar for immune cells. Annu. Rev. Immunol. 2004. 22: 891–928. 15032599 10.1146/annurev.immunol.22.012703.104543

[2] Griffith, J. W. , Sokol, C. L. and Luster, A. D. , Chemokines and chemokine receptors: positioning cells for host defense and immunity. Annu. Rev. Immunol. 2014. 32: 659–702. 24655300 10.1146/annurev-immunol-032713-120145

[3] Nomiyama, H. , Osada, N. and Yoshie, O. , A family tree of vertebrate chemokine receptors for a unified nomenclature. Dev. Comp. Immunol. 2011. 35: 705–715. 21295066 10.1016/j.dci.2011.01.019

[4] Ara, T. , Nakamura, Y. , Egawa, T. , Sugiyama, T. , Abe, K. , Kishimoto, T. , Matsui, Y. and Nagasawa, T. , Impaired colonization of the gonads by primordial germ cells in mice lacking a chemokine, stromal cell‐derived factor‐1 (SDF‐1). Proc. Natl. Acad. Sci. USA 2003. 100: 5319–5323. 12684531 10.1073/pnas.0730719100 PMC154343

[5] Valentin, G. , Haas, P. and Gilmour, D. , The chemokine SDF1a coordinates tissue migration through the spatially restricted activation of Cxcr7 and Cxcr4b. Curr. Biol. 2007. 17: 1026–1031. 17570670 10.1016/j.cub.2007.05.020

[6] Boidajipour, B. , Mahabaleshwar, H. , Kardash, E. , Reichman‐Fried, M. , Blaser, H. , Minina, S. , Wilson, D. et al., Control of chemokine‐guided cell migration by ligand sequestration. Cell 2008. 132: 463–473. 18267076 10.1016/j.cell.2007.12.034

[7] Doitsidou, M. , Reichman‐Fried, M. , Stebler, J. , Koprunner, M. , Dorries, J. , Meyer, D. , Esguerra, C. V. et al., Guidance of primordial germ cell migration by the chemokine SDF‐1. Cell 2002. 111: 647–659. 12464177 10.1016/s0092-8674(02)01135-2

[8] Lapidot, T. , Dar, A. and Kollet, O. , How do stem cells find their way home? Blood 2005. 106: 1901–1910. 15890683 10.1182/blood-2005-04-1417

[9] Zou, Y. , Kottmann, A. , Kuroda, M. , Taniuchi, I. and Littman, D. , Function of the chemokine receptor CXCR4 in haematopoiesis and in cerebellar development. Nature 1998. 393: 595‐599. 9634238 10.1038/31269

[10] Dyer, D. P. , Medina‐Ruiz, L. , Bartolini, R. , Schuette, F. , Hughes, C. E. , Pallas, K. , Vidler, F. et al., Chemokine receptor redundancy and specificity are context dependent. Immunity 2019. 50: 378–389.e375. 10.1016/j.immuni.2019.01.009 PMC6382461 30784579

[11] Schall, T. J. and Proudfoot, A. E. I. , Overcoming hurdles in developing successful drugs targeting chemokine receptors. Nat. Rev. Immunol. 2011. 11: 355–363. 21494268 10.1038/nri2972

[12] Bachelerie, F. , Ben‐Baruch, A. , Burkhardt, A. M. , Combadiere, C. , Farber, J. M. , Graham, G. J. , Horuk, R. et al., International Union of Pharmacology. LXXXIX. Update on the extended family of chemokine receptors and introducing a new nomenclature for atypical chemokine receptors. Pharmacol. Rev. 2014. 66: 1–79. 24218476 10.1124/pr.113.007724 PMC3880466

[13] Bachelerie, F. , Graham, G. J. , Locati, M. , Mantovani, A. , Murphy, P. M. , Nibbs, R. , Rot, A. et al., New nomenclature for atypical chemokine receptors. Nat. Immunol. 2014. 15: 207–208. 24549061 10.1038/ni.2812

[14] Bachelerie, F. , Graham, G. J. , Locati, M. , Mantovani, A. , Murphy, P. M. , Nibbs, R. , Rot, A. et al., An atypical addition to the chemokine receptor nomenclature: IUPHAR Review 15. Br. J. Pharmacol. 2015. 172: 3945–3949. 25958743 10.1111/bph.13182 PMC4543604

[15] Nibbs, R. J. B. and Graham, G. J. , Immune regulation by atypical chemokine receptors. Nat. Rev. Immunol. 2013. 13: 815–829. 24319779 10.1038/nri3544

[16] Antonio Sanchez‐Alcaniz, J. , Haege, S. , Mueller, W. , Pla, R. , Mackay, F. , Schulz, S. , Lopez‐Bendito, G. et al., Cxcr7 controls neuronal migration by regulating chemokine responsiveness. Neuron 2011. 69: 77–90. 21220100 10.1016/j.neuron.2010.12.006

[17] Sierro, F. , Biben, C. , Martinez‐Munoz, L. , Mellado, M. , Ransohoff, R. M. , Li, M. , Woehl, B. et al., Disrupted cardiac development but normal hematopoiesis in mice deficient in the second CXCL12/SDF‐1 receptor, CXCR7. Proc. Natl. Acad. Sci. USA 2007. 104: 14759–14764. 17804806 10.1073/pnas.0702229104 PMC1976222

[18] Ulvmar, M. H. , Werth, K. , Braun, A. , Kelay, P. , Hub, E. , Eller, K. , Chan, L. et al., The atypical chemokine receptor CCRL1 shapes functional CCL21 gradients in lymph nodes. Nat. Immunol. 2014. 15: 623–630. 24813163 10.1038/ni.2889

[19] Graham, G. J. , D6/ACKR2. Front. Immunol. 2015. 6: 280. 26097478 10.3389/fimmu.2015.00280 PMC4456960

[20] Di Liberto, D. , Locati, M. , Caccamo, N. , Vecchi, A. , Meraviglia, S. , Salerno, A. , Sireci, G. et al., Role of the chemokine decoy receptor D6 in balancing inflammation, immune activation, and antimicrobial resistance in Mycobacterium tuberculosis infection. J. Exp. Med. 2008. 205: 2075–2084. 18695004 10.1084/jem.20070608 PMC2526202

[21] Jamieson, T. , Cook, D. N. , Nibbs, R. J. , Rot, A. , Nixon, C. , McLean, P. , Alcami, A. et al., The chemokine receptor D6 limits the inflammatory response in vivo. Nat. Immunol. 2005. 6: 403–411. 15750596 10.1038/ni1182

[22] Lee, K. M. , McKimmie, C. S. , Gilchrist, D. S. , Pallas, K. J. , Nibbs, R. J. , Garside, P. , McDonald, V. et al., D6 facilitates cellular migration and fluid flow to lymph nodes by suppressing lymphatic congestion. Blood 2011. 118: 6220–6229. 21979941 10.1182/blood-2011-03-344044 PMC3234674

[23] Martinez de la Torre, Y. , Locati, M. , Buracchi, C. , Dupor, J. , Cook, D. N. , Bonecchi, R. , Nebuloni, M. et al., Increased inflammation in mice deficient for the chemokine decoy receptor D6. Eur. J. Immunol. 2005. 35: 1342–1346. 15789340 10.1002/eji.200526114

[24] Whitehead, G. S. , Wang, T. , DeGraff, L. M. , Card, J. W. , Lira, S. A. , Graham, G. J. and Cook, D. N. , The chemokine receptor D6 has opposing effects on allergic inflammation and airway reactivity. Am. J. Respir. Crit. Care Med. 2007. 175: 243–249. 17095748 10.1164/rccm.200606-839OC PMC1899265

[25] Hansell, C. A. H. , Fraser, A. R. , Hayes, A. J. , Pingen, M. , Burt, C. L. , Lee, K. M. , Medina‐Ruiz, L. et al., The atypical chemokine receptor Ackr2 constrains NK cell migratory activity and promotes metastasis. J. Immunol. 2018. 201:2510–2519. 30158126 10.4049/jimmunol.1800131 PMC6176105

[26] Nibbs, R. J. , Gilchrist, D. S. , King, V. , Ferra, A. , Forrow, S. , Hunter, K. D. and Graham, G. J. , The atypical chemokine receptor D6 suppresses the development of chemically induced skin tumors. J. Clin. Invest. 2007. 117: 1884–1892. 17607362 10.1172/JCI30068 PMC1904306

[27] Vetrano, S. , Borroni, E. M. , Sarukhan, A. , Savino, B. , Bonecchi, R. , Correale, C. , Arena, V. et al., The lymphatic system controls intestinal inflammation and inflammation‐associated colon cancer through the chemokine decoy receptor D6. Gut 2010. 59: 197–206. 19846409 10.1136/gut.2009.183772

[28] Massara, M. , Bonavita, O. , Savino, B. , Caronni, N. , Mollica Poeta, V. , Sironi, M. , Setten, E. et al., ACKR2 in hematopoietic precursors as a checkpoint of neutrophil release and anti‐metastatic activity. Nat. Commun. 2018. 9: 676. 29445158 10.1038/s41467-018-03080-8 PMC5813042

[29] Lee, K. M. , Danuser, R. , Stein, J. V. , Graham, D. , Nibbs, R. J. and Graham, G. J. , The chemokine receptors ACKR2 and CCR2 reciprocally regulate lymphatic vessel density. EMBO J 2014. 33: 2564–2580. 25271254 10.15252/embj.201488887 PMC4283412

[30] Wilson, G. J. , Hewit, K. D. , Pallas, K. J. , Cairney, C. J. , Lee, K. M. , Hansell, C. A. , Stein, T. and Graham, G. J. , Atypical chemokine receptor ACKR2 controls branching morphogenesis in the developing mammary gland. Development 2017. 144: 74–82. 27888192 10.1242/dev.139733 PMC5278629

[31] McKimmie, C. S. , Singh, M. D. , Hewit, K. , Lopez‐Franco, O. , Le Brocq, M. , Rose‐John, S. , Lee, K. M. et al., G. J., An analysis of the function and expression of D6 on lymphatic endothelial cells. Blood 2013. 121: 3768–3777. 23479571 10.1182/blood-2012-04-425314

[32] Nibbs, R. J. , Kriehuber, E. , Ponath, P. D. , Parent, D. , Qin, S. , Campbell, J. D. , Henderson, A. et al., The beta‐chemokine receptor D6 is expressed by lymphatic endothelium and a subset of vascular tumors. Am. J. Pathol. 2001. 158: 867–877. 11238036 10.1016/s0002-9440(10)64035-7 PMC1850343

[33] Lee, K. M. , Wilson, G. J. , Pingen, M. , Fukuoka, A. , Hansell, C. A. H. , Bartolini, R. , Medina‐Ruiz, L. and Graham, G. J. , Placental chemokine compartmentalisation: a novel mammalian molecular control mechanism. PLoS Biol. 2019. 17: e3000287. 31141500 10.1371/journal.pbio.3000287 PMC6557524

[34] Madigan, J. , Freeman, D. J. , Menzies, F. , Forrow, S. , Nelson, S. M. , Young, A. , Sharkey, A. et al., Chemokine scavenger D6 is expressed by trophoblasts and aids the survival of mouse embryos transferred into allogeneic recipients. J. Immunol. 2010. 184: 3202–3212. 20147628 10.4049/jimmunol.0902118

[35] Hansell, C. A. H. , Schiering, C. , Kinstrie, R. , Ford, L. , Bordon, Y. , McInnes, I. B. , Goodyear, C. S. and Nibbs, R. J. B. , Universal expression and dual function of the atypical chemokine receptor D6 on innate‐like B cells in mice. Blood 2011. 117: 5413–5424. 21450903 10.1182/blood-2010-11-317115 PMC3188399

[36] Ameti, R. , Melgrati, S. , Radice, E. , Cameroni, E. , Hub, E. , Thelen, S. , Rot, A. and Thelen, M. , Characterization of a chimeric chemokine as a specific ligand for ACKR3. J. Leukoc. Biol. 2018. 104: 391–400. 29601107 10.1002/JLB.2MA1217-509R

[37] Ford, L. , Hansell, C. H. and Nibbs, R. B. , Using Fluorescent Chemokine Uptake to Detect Chemokine Receptors by Fluorescent Activated Cell Sorting. In Cardona, A. E. and Ubogu, E. E. (Eds.), Chemokines, Humana Press, Totowa, New Jersey 2013, 203–214. 10.1007/978-1-62703-426-5_13 23625501

[38] Anselmo, A. , Mazzon, C. , Borroni, E. M. , Bonecchi, R. , Graham, G. J. and Locati, M. , Flow cytometry applications for the analysis of chemokine receptor expression and function. Cytometry A 2014. 85: 292–301. 24464630 10.1002/cyto.a.22439

[39] Ford, L. B. , Cerovic, V. , Milling, S. W. , Graham, G. J. , Hansell, C. A. and Nibbs, R. J. , Characterization of conventional and atypical receptors for the chemokine CCL2 on mouse leukocytes. J. Immunol. 2014. 193: 400–411. 24890717 10.4049/jimmunol.1303236 PMC4065784

[40] Le Brocq, M. L. , Fraser, A. R. , Cotton, G. , Woznica, K. , McCulloch, C. V. , Hewit, K. D. , McKimmie, C. S. et al., Chemokines as novel and versatile reagents for flow cytometry and cell sorting. J. Immunol. 2014. 192: 6120–6130. 24850722 10.4049/jimmunol.1303371 PMC4821367

[41] Singh, M. D. , King, V. , Baldwin, H. , Burden, D. , Thorrat, A. , Holmes, S. , McInnes, I. B. et al., Elevated expression of the chemokine‐scavenging receptor D6 is associated with impaired lesion development in psoriasis. Am. J. Pathol. 2012. 181: 1158–1164. 22867710 10.1016/j.ajpath.2012.06.042 PMC3532592

[42] Wigle, J. T. and Oliver, G. , Prox1 function is required for the development of the murine lymphatic system. Cell 1999. 98: 769–778. 10499794 10.1016/s0092-8674(00)81511-1

[43] Paine, R., 3rd , Christensen, P. , Toews, G. B. and Simon, R. H. , Regulation of alveolar epithelial cell ICAM‐1 expression by cell shape and cell‐cell interactions. Am. J. Physiol. 1994. 266: L476–L484. 7909997 10.1152/ajplung.1994.266.4.L476

[44] Castanheira, F. , Borges, V. , Sonego, F. , Kanashiro, A. , Donate, P. B. , Melo, P. H. , Pallas, K. et al., The atypical chemokine receptor ACKR2 is protective against sepsis. Shock 2018. 49: 682–689. 29589840 10.1097/SHK.0000000000000969

[45] Bazzan, E. , Saetta, M. , Turato, G. , Borroni, E. M. , Cancellieri, C. , Baraldo, S. , Savino, B. et al., Expression of the atypical chemokine receptor D6 in human alveolar macrophages in COPD. Chest 2013. 143: 98–106. 22797410 10.1378/chest.11-3220

[46] Cossarizza, A. , Chang, H. D. , Radbruch, A. , Acs, A. , Adam, A. , Adam‐Klages, S. , Agace, W. et al., Guidelines for the use of flow cytometry and cell sorting in immunological studies. Eur. J. Immunol. 2019.49: 1457–1973. 31633216 10.1002/eji.201970107 PMC7350392

