# Supplementary material for: Analysis of lung stromal expression of the atypical chemokine receptor ACKR2 reveals unanticipated expression in murine blood endothelial cells
Source: Eur J Immunol. 2020 Mar 17;50(5):666–75. doi: 10.1002/eji.201948374 (PMC8638673; doi:10.1002/eji.201948374)
Supplement: Supplementary file 1 — Supporting Information [file EJI-50-666-s001.docx]

**Supplemental Figure 1:** summary diagram showing expression analysis from Immgen indicating the strong expression of ACKR2 in mouse lung.

**Supplemental Figure 2:** CCL22 concentrations in the plasma of WT and ACKR2-/- (KO) mice at 4 and 8 weeks of age.

**Supplemental Figure 3:** in silico analysis of the molecular signatures of tissue-specific microvascular endothelial cells for ACKR2 expression. Lung endothelial cells expression is indicated by the arrow.

**Supplemental Figure 4:** Flow cytometry profile showing that selected Prox-1+ve cells in pulmonary digests are exclusively co-positive for CD31 and gp38. (n=3). Upper panels in this figure represent analysis of background signals in control WT mice and the lower panels analysis of Prox1 reporter mice.

**Supplemental Figure 5:** Gating strategies used throughout the study.
